# Comparison of Conventional Hang-Back Technique With Modified (Anchored) Hang-Back in Medial Rectus Recession: A Retrospective Study

**DOI:** 10.7759/cureus.84294

**Published:** 2025-05-17

**Authors:** Munirah Z Aldofyan, Haneen E Alsubki, Ghufran Alarfaj, Maan S Alkharashi

**Affiliations:** 1 Ophthalmology, King Saud University, Riyadh, SAU; 2 Ophthalmology, Dhahran Eye Specialist Hospital, Dhahran, SAU; 3 Ophthalmology, Boston Children’s Hospital, Boston, USA; 4 Ophthalmology, Harvard Medical School, Boston, USA

**Keywords:** bilateral medial rectus recession, esotropia, pediatric ophthalmology & strabismus, strabismus, ‏strabismus, strabismus treatment and surgery

## Abstract

Purpose: To assess the efficacy of the modified hang-back technique (HBT) in comparison to conventional HBT. In this study, we have compared the two techniques in treating esotropia in terms of surgical success, development of consecutive exotropia, and the need for reoperation.

Method:* *A retrospective medical record review of all patients who underwent bilateral medial rectus recession (BMR) between January 2016 and December 2020, with modified HBT or conventional HBT at our institution by four strabismologists. Angles of deviation were measured preoperatively, at one week, at 6-12 weeks, and at final follow-up postoperatively. We evaluated surgical success (alignment within 10 prism diopters [PD] of orthotropia) and complications such as the development of consecutive exotropia and the need for reoperation.

Results:* *The record review identified 103 patients who underwent BMR: 83 in modified HBT vs. 20 in conventional HBT. The overall success rate at final follow-up was similar between the two groups: the success rate in the conventional HBT group was 83.3% (median interquartile range [IQR]) follow-up months 11.0 (6.0-12.8) compared to 72.6% (median IQR) follow-up months 12.0 (12.0-20.0) in the modified HBT group (p=0.435). The rate of consecutive exotropia development was higher in modified HBT (4.83%) vs. 0% in conventional HBT. Reoperation was required for two patients in the modified HBT group (2.4%), both for overcorrection, compared to one patient in the conventional HBT group (5%) for undercorrection (p=0.536).

Conclusion: Conventional HBT is a comparable method to the modified HBT in bilateral medial rectus recession for treating esotropia with similar surgical outcomes.

## Introduction

Rectus muscle recession can be performed using various techniques. These include conventional recession, where the muscle is reattached directly to the sclera with fixed sutures at the desired location; the conventional hang-back technique (HBT), also known as suspension recession, in which the muscle is suspended from its original insertion by a suture loop; and the modified hang-back technique (modified HBT), which is similar to the conventional HBT but includes an additional step of placing a superficial scleral bite at the intended new insertion site before securing the muscle back at its original insertion.

The conventional HBT has gained popularity in recent years due to its ease and efficiency, offering outcomes comparable to traditional recession methods while reducing certain complications, such as globe perforation [1-3]. However, studies have also reported higher rates of overcorrection associated with this technique, likely due to pseudotendon formation [1,4].

To address this limitation, the modified HBT was introduced. Incorporating a scleral anchoring bite potentially improves muscle stability and reduces pseudotendon formation, thus decreasing the risk of consecutive exotropia. Additionally, it may offer more accurate correction in cases with A and V pattern deviations [1].

While several studies have evaluated the outcomes of conventional recession and the conventional HBT [2,3,5,6], limited data exist comparing the modified HBT directly with the conventional HBT. This study aims to compare the clinical outcomes of these two techniques in patients with comitant esotropia, specifically evaluating surgical success (defined as ocular alignment within 10 prism diopters of orthotropia), the incidence of consecutive exotropia, and the need for reoperation.

## Materials and methods

A retrospective medical record review was conducted after approval by the Institutional Review Board (IRB) of King Saud University in Riyadh, Saudi Arabia. The study and data collection conformed to all local regulations and were conducted in accordance with the principles of the Declaration of Helsinki.

Patients included in the study had various types of comitant esotropia requiring surgery. We included all patients diagnosed with comitant esotropia who underwent surgical intervention between January 2016 and December 2020, as long as they met the inclusion criteria and had no exclusion factors. All patients underwent bilateral medial rectus recession performed by four strabismologists: two used the conventional hang-back technique (HBT), and the other two employed the modified hang-back technique (modified HBT). Patients were excluded if the strabismus was restrictive or paralytic in nature, if the patient had nystagmus, if the angle of deviation was 65 prism diopters (PD) or more (as such cases would require three-muscle surgery), if the patient had severe amblyopia (defined as vision ≤ 20/100), if the patient lacked fixation or had eccentric fixation, if anterior or posterior segment pathology was present, if the patient had undergone previous strabismus surgery, if additional procedures such as vertical or oblique muscle surgery were performed simultaneously, or if follow-up duration was less than six months.

Patient demographics, including age and sex, were recorded. Clinical examination focused on visual acuity and angle of deviation at near and distance in PD using the alternate prism-and-cover test (APCT), as well as the indication for surgery and the amount of muscle recession (in millimeters). Postoperative visual acuity, angle of deviation in PD, and any complications were documented at one week, 6-12 weeks, and 6-12 months after surgery. The amount of recession was determined based on the angle of deviation and the dosing tables described by Wright and Hunter, which use equivalent measurements [7,8].

All surgeries were performed under general anesthesia. In the conventional hang-back technique, the conjunctiva was opened via either a fornix or limbal incision. The medial rectus muscle was isolated using a muscle hook and freed from surrounding attachments. A central knot was tied using a double-armed 6-0 polyglactin suture (Vicryl; Ethicon Inc., Johnson & Johnson, Somerville, NJ) near the muscle insertion, with additional locking sutures placed at the edges. The muscle was then detached from the sclera by cutting near the insertion. Sutures were reattached to the sclera at the original insertion site using partial thickness passes, and the muscle was advanced forward. Calipers were used to measure the hang-back distance, and a locking needle holder was placed at the desired recession length. The sutures were tied over the needle holder, which was then removed, allowing the muscle to hang back by the predetermined amount.

The modified hang-back technique (modified HBT) followed a similar procedure; however, after muscle detachment, the desired recession point on the sclera was marked, and superficial partial-thickness bites were taken at that site. The muscle was advanced to rest against these bites, and the sutures were tied at the original insertion to secure the recession. 

Statistical methods used

Data were collected, stored, and managed in a spreadsheet using Microsoft Excel 2010® software (Redmond, USA). Data were analyzed using IBM Corp. Released 2011. IBM SPSS Statistics for Windows, Version 20.0. Armonk, NY: IBM Corp. Tests for normality for the continuous variables were done using the Shapiro-Wilk test and Q-Q plots. The data were not normally distributed and reported as median interquartile range (IQR). Consequently, the Mann-Whitney test was used to test the differences between the conventional hang-back technique (CHBT) and modified hang-back technique (MBHT) groups. Categorical variables are presented as frequencies (percentages), and the chi-squared test is used for the comparison of proportions between the groups. Results were considered statistically significant if p < 0.05.

## Results

In reviewing the patients’ records, 103 patients had bilateral medial rectus recession and met our inclusion criteria. There were 83 (80.6%) patients in the modified hang-back technique (modified HBT) group and 20 (19.4%) in the conventional hang-back technique (HBT) group. All patients included in this study had not previously undergone strabismus surgery. Characteristics of patients in both groups are summarized and compared in Table 1. Notably, there was no statistically significant difference between the two groups in the preoperative angle of deviation. Of all other characteristics, only the gender distribution difference was statistically significant (p=0.002).

**Table 1:** Patient characteristics and preoperative angle of deviation. IQR: interquartile range; *statistically significant at 5% level of significance Data are presented as median (IQR) for continuous variables and frequency (%) for categorical variables. The Mann-Whitney test is used for continuous variables; the chi-square test is used for categorical variables. Due to limitations in retrospective data analysis, test statistics could not be retrieved, but p-values are reported.

Characteristics	Conventional HBT	Modified HBT	P-value
Number of patients, n (%)	20 (19.4)	83 (80.6)	-
Age median (IQR) (years)	6.0 (2.0-15.0)	6.0 (4.0-8.0)	0.860
Male, n (%)	16 (31.4)	35 (68.6)	0.002*
Preoperative angle of deviation at distance, median (IQR)	36.5 (26.3-45.0)	32.0 (25.0-40.0)	0.260
Preoperative angle of deviation at near, median (IQR)	38.0 (30.0-47.3)	35.0 (28.0-45.0)	0.318

The surgical success at the 6-12-week postoperative visit was significantly higher in the conventional HBT group (93.3%) than in the modified HBT group (64.0%) (p=0.028). However, surgical success at final follow-up was not statistically different between the two groups, although there was a trend of more success in the conventional HBT group (83.3%); median IQR follow-up months 11.0 (6.0-12.8) compared to (72.6%); median (IQR) follow-up months 12.0 (12.0-20.0) in the modified HBT group (p=0.435).

In comparing preoperative angles of deviations at distance, the medians in conventional HBT and modified HBT were similar (36.5 and 32.0, respectively). Whereas the angle of deviation at distance postoperatively, the median IQR was significantly lower in the conventional HBT group at one week, 0.0 (0.0-5.0), compared to 5.0 (0.0-11.8) in the modified HBT group (p= 0.007). At the final follow-up, the angle of deviation at distance postoperatively was 0.0 (0.0-5.0) in the conventional group compared to 6.0 (0.0-12.0) in the modified HBT group (p=0.050) (Table 2).

**Table 2:** Postoperative angle of deviation at distance. IQR: interquartile range; *statistically significant at 5% level of significance Data are presented as median (IQR). The Mann-Whitney test was used to compare groups. Test statistic values were not retained in retrospective records; reported p-values are retained for significance reporting.

Postoperative Time	Conventional HBT Median (IQR)	Modified HBT Median (IQR)	P-value (Mann-Whitney)
1 Week Post-op	0.0 (0.0–5.0)	5.0 (0.0–11.8)	0.007*
6–12 Week Post-op	0.0 (0.0–10.0)	5.0 (0.0–16.0)	0.076
Final Follow-up	0.0 (0.0–5.0)	6.0 (0.0–12.0)	0.050

Three patients in the modified HBT group developed consecutive exotropia of an angle more than 10 PD at their final follow-up visit (3/62; 4.83%) compared to none (0/12; 0%) in the conventional HBT group (Table 3).

**Table 3:** Overcorrection vs. undercorrection at the final follow-up visit. Data are presented as frequency (%). The chi-square test was used for between-group comparison. Due to limitations in retrospective data access, test statistics are not available, but p-values are shown.

Correction Type	Conventional HBT	Modified HBT	P-value
>10 PD consecutive exotropia (over-corrected)	0% (0/12)	4.83% (3/62)	-
>10 PD residual esotropia (under-corrected)	16.6% (2/12)	22.5% (14/62)	0.648

Reoperation was performed in two patients in the modified HBT group (2/83, 2.4%), both for overcorrection, compared with one patient in the conventional HBT group (1/20, 5%) for undercorrection (p=0.536). 

## Discussion

Rectus muscle recession using the hang-back technique (HBT) has become increasingly popular among strabismus surgeons, largely due to its advantages over conventional fixed recession techniques. These advantages include a lower risk of globe perforation, easier surgical exposure, and shorter operating times [1,2,4,6]. However, HBT also carries disadvantages, such as pseudotendon formation and uncertainty regarding the final position of muscle reattachment, which may contribute to unpredictable surgical outcomes, particularly overcorrection or undercorrection [1,6].

The modified hang-back technique (HBT) was introduced to address these limitations by adding a superficial scleral anchoring bite at the new muscle insertion site. This theoretically improves muscle stability, reduces pseudotendon formation, and allows more precise correction, particularly in cases with A or V pattern deviations. In our study, however, the modified HBT did not demonstrate clear clinical advantages over the conventional HBT. Surgical success rates at final follow-up were comparable between the two techniques (83.3% vs. 72.6%; p=0.435), and rates of under- and overcorrection were similarly nonsignificant.

Our results are partly consistent with prior research. For example, Andrew K. et al. reported similar success rates between conventional and modified HBT (69.2% vs. 67.4%) [1], and Ameri et al. also found no significant difference in outcomes between the two techniques in a mixed cohort of esotropia and exotropia patients [9]. Similarly, Nabie et al. found equivalent results in exotropia correction using anchored versus conventional HBT [10], suggesting that the theoretical biomechanical advantage of the anchoring step may not consistently translate into superior clinical outcomes across all populations.

Interestingly, our findings diverged from those of Andrew K. et al. regarding consecutive exotropia. While they reported significantly higher consecutive exotropia rates in the conventional HBT group (20.5%) compared to the modified HBT group (4.7%) (P = 0.031), our study found a higher rate of consecutive exotropia in the modified HBT group (4.83%) and none in the conventional group. This discrepancy may reflect differences in patient selection, surgical technique nuances, case complexity, or follow-up duration. For instance, larger or more complex deviations in the modified HBT group may have increased the risk of overcorrection despite the additional anchoring step. Additionally, variation in surgical expertise or tissue healing responses may explain differences in pseudotendon formation and muscle stability between studies.

Regarding undercorrection, Andrew K. et al. reported significantly higher undercorrection rates in the modified HBT group (27.9%) compared to the conventional HBT group (10.4%) (p=0.045), whereas we observed no statistically significant difference (22.5% vs. 16.6%; p=0.648). This again highlights the multifactorial nature of surgical outcomes in strabismus correction and suggests that the modified technique does not uniformly reduce the risk of undercorrection.

Notably, none of our patients developed serious complications such as globe perforation or retinal detachment, which aligns with previous reports supporting the overall safety of both techniques when performed carefully [1,2,10]. However, it is important to acknowledge that the modified HBT increases operative time and technical demands and eliminates the option of using adjustable sutures, an important tool in selected cases. This may limit its adoption, particularly among less experienced surgeons or in training environments.

Overall, our findings contribute to the growing body of evidence suggesting that, while the modified HBT offers theoretical advantages, its routine use may not result in superior clinical outcomes compared to the conventional HBT. Future research, ideally through large-scale, prospective, multicenter trials with standardized surgical protocols and longer follow-up, is needed to better define the indications, benefits, and limitations of each technique.

Study limitations

While this study is limited by its retrospective design and the unequal group sizes, these aspects were addressed by applying strict inclusion and exclusion criteria to ensure a well-defined, homogenous cohort and to minimize potential confounding factors. The smaller sample size in the conventional HBT group reflects real-world surgical distribution patterns. Nevertheless, meaningful comparisons were still possible through appropriate statistical methods.

We acknowledge that this group size imbalance may have limited the statistical power to detect subtle differences between the groups. Therefore, some nonsignificant results should be interpreted with caution, as the study may have been underpowered to reveal small but clinically relevant effects. Despite this, the standardized outcome definitions and consistent follow-up protocols add robustness to the findings. Future prospective, randomized studies with larger and more balanced cohorts are recommended to validate and expand upon these results.

## Conclusions

This study demonstrates that both the conventional hang-back technique and modified hang-back techniques provide reliable options for bilateral medial rectus recession in esotropia. Surgical outcomes were similar, indicating that either method can be effectively used in clinical practice. Technique selection should be based on the surgeon's expertise and individual patient characteristics.
